# A molecular signature in blood identifies early Parkinson’s disease

**DOI:** 10.1186/1750-1326-7-26

**Published:** 2012-05-31

**Authors:** Leonid Molochnikov, Jose M Rabey, Evgenya Dobronevsky, Ubaldo Bonuccelli, Roberto Ceravolo, Daniela Frosini, Edna Grünblatt, Peter Riederer, Christian Jacob, Judith Aharon-Peretz, Yulia Bashenko, Moussa BH Youdim, Silvia A Mandel

**Affiliations:** 1 Sackler School of Medicine, Tel Aviv University, Tel Aviv, Israel; 2 Asaf HaRofeh Medical Center, Department of Neurology, Zerifin, Israel; 3 Department of Neuroscience, University of Pisa, Pisa, Italy; 4 Neurobiochemistry Laboratory, Department of Child and Adolescent Psychiatry, University Zurich- Irchel, Zurich, Switzerland; 5 Department of Psychiatry, Psychosomatic and Psychotherapy, University Hospital of Würzburg, Würzburg, Germany; 6 Department of Neurology, Rambam Medical Center, Haifa, Israel; 7 Technion-Faculty of Medicine, Eve Topf Center for Neurodegenerative Diseases Research, Department of Molecular Pharmacology, P.O.B. 9697 31096, Haifa, Israel; 8 Department of Biology, Yonsei Central University, Seoul, Republic of Korea

**Keywords:** Alzheimer’s disease, Sporadic Parkinson’s disease, Blood Biomarker, CSF Biomarkers, E3 ubiquitin ligase, SCF, SKP1, Heat shock protein Hsc-70, Early diagnosis

## Abstract

**Background:**

The search for biomarkers in Parkinson’s disease (PD) is crucial to identify the disease early and monitor the effectiveness of neuroprotective therapies. We aim to assess whether a gene signature could be detected in blood from early/mild PD patients that could support the diagnosis of early PD, focusing on genes found particularly altered in the substantia nigra of sporadic PD.

**Results:**

The transcriptional expression of seven selected genes was examined in blood samples from 62 early stage PD patients and 64 healthy age-matched controls. Stepwise multivariate logistic regression analysis identified five genes as optimal predictors of PD: p19 S-phase kinase-associated protein 1A (odds ratio [OR] 0.73; 95% confidence interval [CI] 0.60–0.90), huntingtin interacting protein-2 (OR 1.32; CI 1.08–1.61), aldehyde dehydrogenase family 1 subfamily A1 (OR 0.86; 95% CI 0.75–0.99), 19 S proteasomal protein PSMC4 (OR 0.73; 95% CI 0.60–0.89) and heat shock 70-kDa protein 8 (OR 1.39; 95% CI 1.14–1.70). At a 0.5 cut-off the gene panel yielded a sensitivity and specificity in detecting PD of 90.3 and 89.1 respectively and the area under the receiving operating curve (ROC AUC) was 0.96.

The performance of the five-gene classifier on the *de novo* PD individuals alone composing the early PD cohort (n = 38), resulted in a similar ROC with an AUC of 0.95, indicating the stability of the model and also, that patient medication had no significant effect on the predictive probability (PP) of the classifier for PD risk. The predictive ability of the model was validated in an independent cohort of 30 patients at advanced stage of PD, classifying correctly all cases as PD (100% sensitivity). Notably, the nominal average value of the PP for PD (0.95 (SD = 0.09)) in this cohort was higher than that of the early PD group (0.83 (SD = 0.22))*,* suggesting a potential for the model to assess disease severity. Lastly, the gene panel fully discriminated between PD and Alzheimer’s disease (n = 29).

**Conclusions:**

The findings provide evidence on the ability of a five-gene panel to diagnose early/mild PD, with a possible diagnostic value for detection of asymptomatic PD before overt expression of the disorder.

## Background

Currently, the diagnosis of Parkinson’s disease (PD) is based mainly on clinical criteria [1]. In addition the evaluation of the clinical status and evolution of PD are based on examination of symptoms, utilizing structured scoring systems (Unified Parkinson’s Disease Rating Scale, (UPDRS) [2], Short Parkinson Evaluation Scale, (SPES), SCales for Outcomes in PArkinson’s diseases– (SCOPA) [3,4] and the Hoehn and Yahr (H&Y) staging scale [5]. Although PD can be accurately diagnosed in patients with a typical presentation and positive response to levodopa with a sensitivity of 93% [6], differential diagnosis from other entities presenting parkinsonism (e.g. essential tremor, progressive supranuclear palsy (PSP), multisystem atrophy (MSA), corticobasal degeneration (CBD)) may be challenging. Imaging studies using positron emission tomography (PET) with ^18^ F]-Dopa, single photon emission tomography (SPECT) with ^123^I]-β-CIT or diffusion-weighted MRI could improve differential diagnosis of parkinsonism [7-9], but cost-effectiveness remains a problem.

Furthermore, these tools do not provide a specific and sensitive enough PD diagnosis [10]*.* The discovery of mutations linked to familial PD and the implementation of microarray-based gene expression profiling during the past decade, has provided additional clues for the pathophysiology of sporadic PD as well as potential molecular targets that may be of relevance to the disease [11-16]. Our previous gene expression study conducted in post-mortem substantia nigra (SN) obtained from sporadic PD patients identified a cluster of genes that were most differentially expressed in sporadic parkinsonian SN, by a factor of ≥1.5, compared to non-diseases controls [11]. The transcripts were mainly related to DA transmission and metabolism, and protein handling/degradation mechanisms previously known to be involved in the pathophysiology of the disease. Examples include *SKP1A* (p19, S phase kinase-associated protein 1A), a component of the largest class of E3 ubiquitin ligases, SCF (Skp1, Cullin 1, a substrate recognizing F-box protein and Rbx1) [17,18], *HSPA8* (heat shock 70-kDa protein 8, encoding chaperone Hsc-70) [19], and 19 S proteasomal protein *PSMC4*/*S6b*/*TBP7*, whose levels were decreased in PD. Also, aldehyde dehydrogenase family 1, subfamily A1 (*ALDH1A1*) involved in the degradation of aldehyde derivatives of DA, and vesicular monoamine member 2 (VMAT2) were down-regulated.

Recent studies have shown the feasibility of studying peripheral (cerebrospinal fluid (CSF), blood and urine) signatures or biomarkers for potential diagnosis and early detection of PD [20] such as alpha-synuclein and DJ-1 protein in the CSF [21-23]. Serum uric acid appears to be the first molecular factor linked to a decreased risk of PD [24,25] and to inversely correlate with clinical and radiographic progression of typical PD [26]. Furthermore, increasing evidence indicates that peripheral tissue shares significant protein/gene expression similarities to inaccessible central nervous system (CNS) tissues [27,28] and thus may offer valuable surrogate markers for neuropsychiatric disorders. For instance, a recent large serum proteomic study with psychiatric patients has identified a number of proteins belonging to pathways previously shown to be involved in the pathophysiology of either depression or schizophrenia, such as growth factors, cytokines and neurotrophins [29]. In a microarray gene profiling study with blood PD tissue, it was demonstrated a panel of genes associated with PD risk, some of them involved in pathobiologically relevant disease processes of the ubiquitin– proteasome pathway system (UPS), mitochondrial function, and apoptosis [27]. More recently, a genome-wide pathway meta-analysis (meta-GSEA) with PD tissues has particularly identified a set of genes controlling cellular bioenergetics and mitochondria biogenesis that were shared by both brain and blood [30]. Using a similar, but less comprehensive approach of integrating openly available and new PD microarray data, a panel of genes was identified to be commonly expressed in brain and blood samples [31]. These findings suggest that blood and brain neuronal cells might have a common regulatory mechanism for gene expression.

The seven genes chosen for the study form part of the core of 20 gene transcripts most significantly altered in PDSN from sporadic PD patients [11]. Here we analyze their expression in peripheral blood from early PD patients to identify a signature that could support the diagnosis of the disease.

## Results

### Identification of a PD risk gene signature

**Figure 1:**
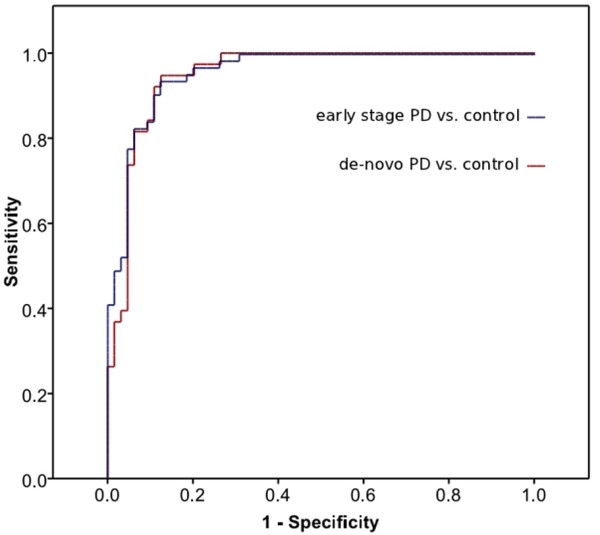
**The receiver operating characteristic curve (ROC) of the logistic regression model for discriminating between early PD and control.** The blue line depicts integrative specificity and sensitivity for the PP derived from five variables (SKP1A, HIP2, ALDH1A1, PSMC4 and HSPA8 transcriptional expression levels, AUC = 0.96) of the early/mild PD *vs* healthy control subject cohorts. At a cut-off point of 0.5 it was possible to distinguish between PD individuals and healthy controls with sensitivity and specificity values of 90.3% and 89.1% respectively. The red line shows the performance of the classifier on the 38 *de novo*, non medicated PD individuals alone from the early PD cohort, which resulted in a similar ROC with an AUC of 0.95, indicating the stability of the logistic model.

A five-gene panel was found that optimally discriminates early PD from controls based on stepwise multivariate logistic regression analysis of seven genes that were found significantly altered in sporadic PD SN tissue [11]*(ALDH1A1PSMC4SKP1AHSPA8*, c-src Tryosine Kinase *CSK*, huntingtin interacting protein 2/ubiquitin-conjugating enzyme E2K *HIP2* and Egl nine homolog 1 *EGLN1*). The composition of the PD cohort comprised mild/early stage PD (38 *de novo* and 24 medicated PD, H&Y = 1.40 (SD = 0.56))*.* As shown in Table 1*SKP1A, HIP2, ALDH1A1, PSMC4* and *HSPA8,* were classified as optimal predictors for PD risk. Negative regression coefficients (B) indicate an inverse relationship between transcript expression and risk for PD. Thus, the negative values of *ALDH1A1PSMC4* and *SKP1A* suggest that these genes possibly decrease the risk for the occurrence of PD with OR values of 0.86, 0.73 and 0.73 respectively, whereas *HSPA8* and *HIP2* significantly increase the risk for PD, with OR values of 1.39 and 1.32, respectively. The predicted probability (PP) for PD in a tested individual was calculated by the equation described in the Materials and Methods and the diagnostic performance of the gene cluster was assessed by a receiver operating characteristic curve (ROC), showing high sensitivity and specificity for the early stage PD group versus healthy controls at various cut-offs (Figure 1, blue line), with an area under the curve (AUC) of 0.96. The performance of the classifier on the 38 *de novo*, non medicated PD individuals alone from the early PD cohort, resulted in a similar ROC with an AUC of 0.95, indicating the stability of the classifier model (Figure 1, red line) and suggesting that medication does not influence the predictive value of the genetic signature. In support, no significant difference was observed between the PP average value of the non-medicated, *de-novo* PD cohort (0.81 (SD = 0.20)) and that of the early medicated population (0.87 (SD = 0.25); *t*-test, p = 0.354).

**Table 1:** Variables in the predicted probability equation

	** B**	**P value**	**OR**	**95%C.I. for OR**	
				**Lower**	**Upper**
L_ SKP1	−0.313	0.003	0.731	0.595	0.898
L_ HIP2	0.274	0.008	1.315	1.076	1.608
L_ALDH1A1	−0.148	0.030	0.862	0.754	0.986
L_ PSMC4	−0.318	0.002	0.727	0.595	0.889
L_ HSPA8	0.330	0.001	1.391	1.139	1.699

*L*_ Natural logarithm.

*B*_ Regression coefficient. Negative values indicate an inverse relationship between transcript expression and risk for PD and the opposite for positive values.

*OR*_odds ratio.

*C.I.*_Confidence Interval.

Given that L_ and not raw ratios were used in logistic regression analysis, L_values were multiplied by 10 to avoid skewed OR values.

**Figure 2:**
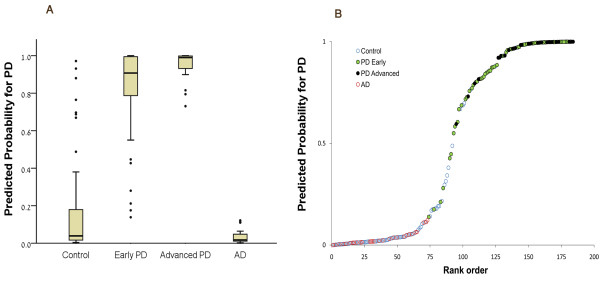
**Predictive probability (PP) for PD in early PD subjects compared to advanced PD, AD and healthy control groups. a**) The distribution of the PP values of the early/mild PD, advanced PD, AD and healthy cohorts derived from the logistic regression analysis are depicted. The box plots represent 50% of the cases, with the median (horizontal bold line) and the 1st and 3rd quartile values (bottom and top of the box, respectively). The bottom and top whiskers show the lowest and the highest datum within 1.5x interquartile range (IQR, the range between the 1^st^ and 3^rd^ quartile) from bottom and top of the box, respectively. Outliers are denoted by black dots. Beyond the cut-off of PP = 0.5 the subjects were considered as having PD. **b**) The performance of the classifier across the entire data set. Blood samples are ordered by their PP for PD. 86 out of 93 individuals that ranked in the upper panel (>0.5) are PD. 86 out of 92 individuals that ranked in the lower panel (< 0.5) are controls. PD = Parkinson’s disease. AD = Alzheimer’s disease.

The distribution of the PP values of the early/mild PD cohort *vs* those of healthy (control) subjects is depicted in Figure 2A. To better represent the true predictive value of the model, we selected a cut-off of 0.5 beyond which the subjects were considered as having PD. At this cut-off point we were able to distinguish between PD individuals and healthy controls with sensitivity and specificity values of 90.3% and 89.1% respectively.

Demographic analysis revealed no significant difference in age between the early PD group and control group (*t* test, p = 0.382; see Table 2 for patients details). When age was introduced as a possible explanatory variable within the regression model which included the gene expression variables, it had no impact on the PP of the model for PD. Regarding gender, although the proportion of males was significantly higher in the early PD group, a two-way ANOVA, with gender (male/female) and group (control/PD) as variables, revealed that the differential gene expression resulted from the group variable only (p < 0.001), being independent of gender (p = 0.522) or gender/group interaction (p = 0.346).

**Table 2:** Demographics and Hoehn & Yahr scores

**Diagnostic groups**	**Control**	**PD (total cases)**	**PD Early Stage**	**PD Advanced Stage**	**AD**
n	64	92	62	30	29
Age (SD)	65.9 (7.9)	65.52 (10.11)	64.5 (10.2)	67.67 (9.7)	73.0 (8.4)
Minimum Age	52	35	35	52	58
Maximum Age	82	88	88	88	87
Gender (% of males)	43.8	67.4	66.1	70.0	44.8
Hoehn & Yahr (SD)	0	1.95 (1.02)	1.40 (0.56)	3.07 (0.8)	0

### Validation of specificity and sensitivity of the gene risk panel

To validate the diagnostic value of the PD gene panel, a separate cohort of 30 PD patients at advanced disease stage and 29 patients with Alzheimer’s disease (AD) were tested with the logistic classification model obtained from the early PD-healthy control samples. The gene cluster positively classified all 30 cases as PD (100% sensitivity) and discriminated PD from AD with 100% specificity (all 29 cases were classified as non-PD), thus supporting the diagnostic value of the molecular signature for detecting PD (Figure 2A). Notably, the nominal average value of the PP for PD in late- stage cohort (0.95 (SD = 0.09); H&Y: 3.07 (SD = 0.81)) was higher than that of the early PD group (0.83 (SD = 0.22); H&Y: 1.40 (SD = 0.56))*,* suggesting a potential for the model to assess disease severity. The performance of the classifier across the entire data set is depicted in Figure 2B. 86 out of 93 individuals that ranked above a PP of 0.5 are PD. 86 out of 92 individuals that ranked below a PP of 0.5 are controls.

### Relative transcript expression in the different cohorts

**Figure 3:**
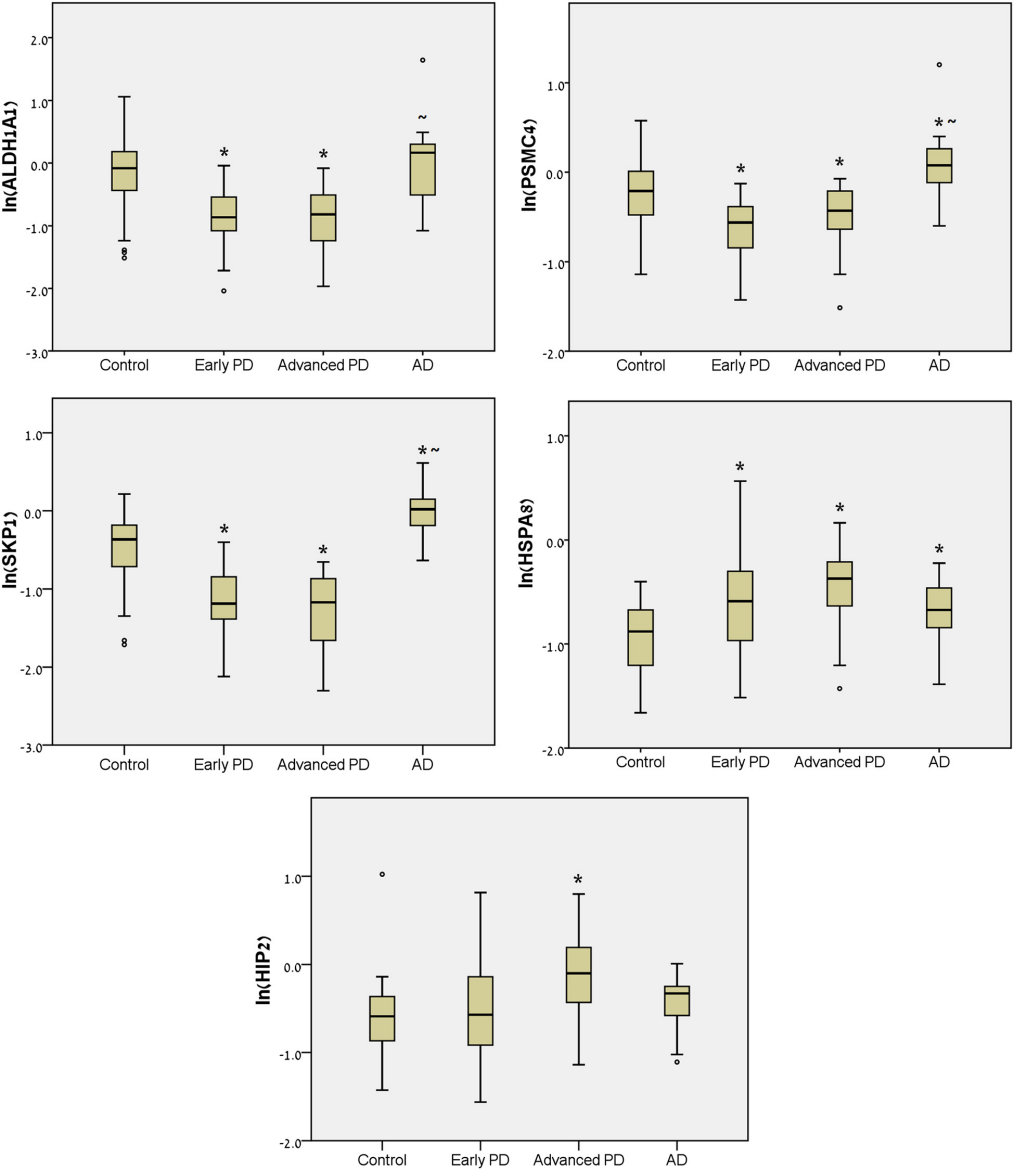
**QRT-PCR assessment of the relative transcript expression levels in the marker panel.** The box plots depict the natural logarithms of the relative gene expression levels (calculated by dividing the QRT-PCR values by the geometric mean of the HKs ACTB, ALAS1 and GAPDH raw quantities) for the individual five genes in blood samples of 62 early stage PD patients (Early PD; H&Y = 1.40 (SD = 0.56)), 30 PD patients at relatively advanced stage (Advanced PD; H&Y = 3.07 (SD = 0.81)), 29 AD patients and 64 healthy age-matched subjects (Control). Outliers are denoted by black dots. The significance was calculated by one-way ANOVA, with post-Hoc Tukey analysis. *p<0.05 *vs* control; ~p<0.05 *vs* early PD. In the text, expression level changes (percentage) refer to relative gene expression levels and not to the natural logarithms.

Figure 3 shows the differential transcription pattern of the individual five genes composing the panel, in the four cohorts of subjects: healthy control, early stage PD, advanced stage and AD. Prominent transcript level reductions in *ALDH1A1*, *PSMC4* and *SKP1A* and a significant elevation in *HSPA8* were seen in the PD groups, compared to healthy controls, as revealed by one-way ANOVA. On the other hand, no significant gene alterations were encountered in *HIP2* in early PD compared to control, whereas a clear increase was seen in advanced PD stage. A different expression pattern was seen in the AD group, supporting the specificity of the gene changes to PD.

The correlative analysis of the expression levels of all the tested transcripts in the control cohort, revealed a significant association between *SKP1A*, *HIP2, ALDH1A1* and *PSMC4* (Table 3). *SKP1A* showed a weaker but significant correlation with two additional transcripts, *HSPA8* and *EGLN1*. In contrast to the findings in the control group, the association of SKP1A with the other transcripts was disrupted in early PD, suggesting a possible functional connection between the panel genes.

**Table 3:** Correlations between the natural logarithms of the relative gene expression levels in the control group

	**HIP2**	**ALDH1A1**	**PSMC4**	**HSPA8**	**CSK**	**EGLN1**
**SKP1A**	R = 0.440**	R = 0.600**	R = 0.480**	R = 0.288*	R = −0.217	R = 0.283*
	p < 0.001	p < 0.001	p < 0.001	p = 0.021	p = 0.196	p = 0.023
**HIP2**	-	R = 0.459**	R = 0.582**	R = 0.296*	R = −0.265	R = 0.371*
		p < 0.001	p < 0.001	p = 0.017	p = 0.112	p = 0.03
**ALDH1A1**		-	R = 0.385**	R = 0.235	R = −0.060	R = 0.299*
			p = 0.002	p = 0.061	p = 0.724	p = 0.016
**PSMC4**			-	R = 0.402**	R = 0.052	R = 0.286*
				p = 0.001	p = 0.761	p = 0.022
**HSPA8**				-	R = 0.237	R = 0.185
					p = 0.158	p = 0.144
**CSK**					-	R = 0.112
						p = 0.508

R = Pearson correlations coefficient.

* P < 0.05; ** P < 0.01.

## Discussion

The results of this study support our hypothesis that there are blood gene biomarkers that can distinguish early PD patients from normal control subjects. Notably, 38 out of the 62 Parkinson cases in the mild/early cohort were *de novo* and so, not treated with any antiparkinsonism drug when the blood samples were obtained while the rest were collected during the first year of medication. This suggests that the genetic signature could be an early diagnostic marker for PD. In support, the classifier model performed equally well in early stage *de novo* PD samples, producing a similar ROC AUC value to that obtained with the entire early PD cohort (de novo and medicated), indicating that patient medication had no significant effect on the PP of the classifier for PD risk and that the model is stable throughout the two PD groups. Supporting this concept, it was recently shown in a population of asymptomatic LRRK2 mutation carriers, that reduced CSF amyloid β and tau species correlated with lower striatal dopaminergic function as determined by PET [32], suggesting that they may serve as potential biomarkers even in asymptomatic phases of the disease. The performance of the gene model was validated in an independent cohort of patients at advanced PD stage where all individuals were correctly classified as PD, while it fully discriminated PD from a group of individuals affected with AD (considered the most common neurodegenerative disease). Giving that misdiagnosis occurs normally at the initial PD stage, the 100% sensitivity obtained with the long-term PD cohort support the feasibility of the biomarker panel to differentiate with certainty between PD and non-PD. Further studies will determine the ability of the panel to differentially diagnose idiopathic PD from patients with other forms of Parkinsonism, such as PSP and MSA.

One main challenge in the development of biological markers is to minimize the number of genes in the classification model while still achieving a high classification rate. The present biomarker signature identified a minimal set of transcripts in blood that has a high discriminating power to categorize the PD early group and to positively/negatively classify the advanced PD and AD cohorts.

A model with fewer genes is likely to yield better generalization (less number of free variables) and optimization of diagnosis. We have found that five out of the seven gene transcripts previously reported to have been changed in sporadic PDSN [11], were found altered in blood of mild/early PD. Our findings argue in support of the view that changes in peripheral blood may have relevance to mechanisms occurring in brain of PD patients and indicate that at least some of the gene expression alterations occurring in PD are not exclusive to the brain, but are expressed also in peripheral blood tissue. Indeed, a large proportion of the genes encoded in the human genome have detectable levels of transcripts in circulating blood cells [33]; When coming into contact with brain tissue, circulating blood cells may provide information concerning the pathological environment of the PD brain.

Gene expression correlation analysis indicates a significant association in blood from healthy control individuals between *SKP1A* and five gene transcripts: *HIP2, ALDH1A1PSMC4HSPA8* and *EGLN1*, while it was absent in early PD, suggesting a functional coordinative role for Skp1. Skp1 takes part in the ubiquitin-proteasome/E3-ligase SCF complex, acting in a module-like manner: Skp1 can interact with several F-box proteins, which play an indispensable role in the selection of target proteins for degradation [17]. Thus, a reduced activity of Skp1 may play a role in the development of PD by impairing the timely degradation of a wide array of proteins, promote their deposition and affect the function of dopaminergic (DAergic) neurons. Skp1, together with the chaperone Hsc-70 encoded by *HSPA8*, the proteasomal ATPase subunit PSMC4, the EGLN1-encoded prolyl hydroxylase and the huntingtin-interacting protein Hip2, are intimately connected to processing/degradation of proteins by UPS/lysosomal- mediated degradation [17-19,34-37]. Further evidence for a possible functional connection between the panel genes is provided by our recent finding showing that silencing *SKP1A* in the SN-derived murine cell line SN4741 induced a parallel down-regulation in the transcripts of *ALDH1A1* and *HSPA8*[38]. Aldh1 was found to be expressed highly and specifically in DA cells of the SN and ventral tegmental area (VTA) [39] having a role in the neutralization of toxic aldehyde derivatives of DA [34]. These highly reactive, neurotoxic aldehydes can accumulate in case of decreased levels of Aldh1, as occurs in SNpc of PD [39,40], and can promote neuronal death. The fact that the five genes comprising the signature, as a group, play important roles in PD neuropathology and are significantly correlated in blood form healthy subjects, add a biological significance to the findings.

Supporting the rationale of identifying molecular changes in peripheral blood that may respond to the pathology in the brain of sporadic PD, Grunblatt et al. [41] recently reported a cluster of four genes in blood tissue that discriminated between PD and healthy controls. One of them, *ALDH1A1* was also detected in our gene signature, independently confirming part of our results. Further support comes from Scherzer et al. [27] who demonstrated a panel of eight genes involved in relevant PD processes such as the UPS, mitochondrial function and apoptosis in whole blood tissue from a heterogeneous cohort of relatively early-staged PD individuals, that correlated with PD risk. It is worth noting that despite the difference in the study design, e.g., the use of large-scale microarrays comprising the whole genome, the restricted eight-gene signature included *HIP2*, also found by us, as a surrogate for PD. In our study, we have performed multi-step logistic regression analysis, which is commonly applied in biomarker research. This procedure recruits in each step the most significant gene discriminating between PD and control in relation to the prior step, thus taking into consideration the cumulative impact of the gene group on the PD risk. In Scherzer’s study, the genes were individually rank-ordered according to the absolute value of their correlation coefficient with PD, disregarding the correlation between their expression levels.

Another major discovery of this investigation is that the PP values of the five-gene signature were accentuated in patients at late PD stage, suggesting a potential for the model to assess disease severity. One relevant point is what could be the biological meaning of this observation. It can be conjectured that the peripheral gene transcriptional changes may reflect evolution of pathogenic processes during PD progression. In analogy, Shi et al. [42] have described a panel of seven CSF proteins that could aid in PD diagnosis and differential diagnosis. Among these, an increase in CSF fractalkine, along with decreased Aβ1–42 levels, correlated with a higher UPDRS score in cross-sectional samples and in a set of longitudinally collected PD samples from the DATATOP study.

When examining the relative quantity of each gene individually at the cross-sectional level, we demonstrated a similar transcriptional pattern for *SKP1A, ALDH1A*, *PSMC4* and *HSPA8* in the two PD cohorts compared to normal controls or AD groups, suggesting that these transcripts are altered at early stages of the disease and not affected by disease progression. However, at this stage, we cannot determine whether the selective elevation of *HIP2* demonstrated only in PD patients at advanced stage of disease, can reflect a disease evolution. Despite the strength of the present findings, there are some issues yet to be addressed. At this point the cross-sectional nature of this study does not allow making a correlation between gene expression and clinical symptoms that may point to the clinical state. Longitudinal studies will establish whether the gene panel can serve as a marker for PD risk or its progression. Although we have initially focused on seven out of the 20 gene transcripts most altered in sporadic PD brains, it is likely that the other risk genes could be also relevant.

## Conclusions

Our current pilot study demonstrated that the blood gene model has strong predictive value for PD diagnosis and possibly may help to identify individuals at presymptomatic stages (patients with depression, sleep disturbances or hyposmia or patients carrying genetic risk factors) who are good candidates for neuroprotective treatment. Such a biomarker will be of value for identification of a pathophysiological subgroup of PD patients that may respond favorably to agents targeting the mechanisms reflected by the gene panel.

Large-scale, prospective, controlled studies, which combine our methodology with quantification of CSF total/oligomers of α-synuclein or/and DJ-1 and brain imaging may be useful as a multi-modal biomarker, not only for early diagnosis but for evaluation of disease progression.

## Methods

### Study population

The subjects examined gave written informed consent according to the ethical committee of each hospital engaged in the study. 185 individuals were enrolled for blood sample mRNA extraction: 62 early/mild PD patients (38 *de novo*, non medicated PD and 24 early PD patients within first year of medication (Hoehn and Yahr; H&Y 1.40, SD = 0.56), 30 PD patients with advanced disease (H&Y 3.07, SD = 0.8), 29 patients with AD (Mini-Mental State Exam = 19.0, SD = 2.73) and 64 healthy age-matched controls without personal or family history of neurodegenerative diseases. For this multi- center, international study blood samples were recruited from the following hospitals: the Department of Neuroscience, University of Pisa (Italy), Hospital of Viareggio (Italy), University Hospital of Würzburg (Germany), Assaf Harofe and Rambam Medical Centers (Tel Aviv and Haifa, Israel). PD patients that met modified United Kingdom Parkinson’s Disease Society Bank Brain clinical diagnostic criteria [1] were diagnosed by neurologists trained in movement disorders. Patient data (age, gender, PD severity score, H&Y, blood count and medication) were registered. Patients with probable AD were recruited by the Clinic for Psychiatry, Psychosomatic and Psychotherapy, University of Würzburg, Assaf Harofe and Rambam Medical Centers. The AD samples from University of Würzburg (n = 10) are part of a study published earlier [41]. All patients met the National Institute of Neurological and Communicative Disorders and Stroke-Alzheimer’s Disease and Related Disorders Association diagnostic criteria [43]. Control blood samples consisted of healthy age- matched subjects that accompanied neurological patients during the visits to the movement disorders centers. The proportion of males in the healthy population was 43.8% with a mean age of 65.9 ± 7.9 and in the PD group (early and advanced) was 67.4% and a mean age of 64.5 ± 10.2 (see Table 2). Total white blood cells count, as well as differential blood cell counts were examined for any bias in gene expression changes. No significant variations were observed via one-way analysis of variance (ANOVA) between PD and healthy control groups in all counts as shown in Additional file 1, Table S1).

### Isolation of total RNA from blood samples and quality control

Venous blood samples were collected using *PAXgene Blood RNA System* Tubes (Becton Dickinson GmbH, Heidelberg, Germany) at the different centers and shipped to the Eve Topf Center in Haifa for RNA extraction and real-time PCR (QRT-PCR) quantification, except for the 10 AD sample cases from University of Würzburg, which were shipped as lyophilized RNA instead of blood tubes. The blood samples were frozen at −80°C until processed for total RNA isolation. Both controls and cases samples were processed in parallel. Total RNA was extracted from whole blood with the *PAXgene™* Blood RNA Kit 50 (PreAnalytiX, Qiagen and BD, Germany). RNA quality was determined spectrophotometrically by NanoDrop 1000 Spectrophotometer (Thermo Fisher Scientific Inc, Wilmington, DE, USA) and by using the Experion^TM^ Automated Electrophoresis System (Bio-Rad Laboratories, Hercules, CA, USA). A representative test from arbitrarily selected RNA samples showing the analysis of the 28 S and 18 S bands is provided in Additional file 2. RNA samples that adhered to quality control criteria (Additional file 1, Methods) were taken for further analysis.

### Quantitative real-time RT-PCR (QRT-PCR)

Total RNA from each blood sample was reversed transcribed employing the *High-Capacity cDNA Reverse Transcription* Kit (Applied Biosystems, Foster City, CA, USA). QRT-PCR was performed using SYBR Green detection chemistry, in the ABI PRISM 7000 Real-Time Sequence Detection System (Applied Biosystems, Foster City, CA, USA, see details in Additional file 1, Methods). Oligonucleotide primers are depicted in Table 4. Gene expression values were normalized to three housekeeping genes (Additional file 1, Methods and Table S2).

**Table 4:** QRT-PCR oligonucleotide primers

**Gene Name**	**Acc number**	**Symbol**	**Catalog No.**
Egl nine homolog 1	NM_022051	EGLN1	QT01021454
Heat shock 70 kDa protein 8	NM_006597, NM_153201	HSPA8	QT00030079
Proteasome (prosome, macropain) 26 S subunit, ATPase 4	NM_006503	PSMC4	QT00035511
Clathrin, light polypeptide	NM_001834	CLTB	QT00081872
Aldehyde Dehydrogenase 1 Family, member A	NM_000689	ALDH1A1	QT00013286
S-phase kinase-associated protein 1A	NM_006930	SKP1	QT00040320
Huntingtin interacting protein 2/ubiquitin-conjugating enzyme E2K	NM_005339, NM_001111113	HIP2=UBE2K	QT00010276
c-src Tryosine Kinase	NM_004383, NM_001127190	CSK	QT00999131
*Housekeeping genes*			
Actin B	NM_001101	ACTB	QT00095431
Aminolevulinate, delta-, synthase 1	NM_000688, NM_199166	ALAS1	QT00073122
Glyceraldehyde-3-phosphate dehydrogenase	NM_002046	GAPDH	QT01192646
Peptidylprolyl isomerase A (cyclophilin A)	NM_021130	PPIA	QT01866137
Ribosomal protein L13A	NM_012423	RPL13A	QT00089915

### Building a risk marker profile

In order to establish a molecular risk marker for PD, a logistic regression model was built via stepwise multivariate logistic regression analysis of the natural logarithms (ln) of the relative gene expression for all seven genes, comparing the PD early/mild subjects (38 *de novo*, non medicated PD and 24 early PD patients) and the healthy control subjects (64 healthy age-matched controls without personal or family history of neurodegenerative diseases).

Step 1. The relative gene expression was calculated by dividing the QRT-PCR values of the seven genes by the geometric mean of the three most stable housekeeping genes expression levels (GAPDH, ACTB and ALAS1).

Step 2. The values were transformed to ln to enable normal distribution.

Step 3. The model was built by progressively adding the variables (relative gene expression) with the lowest, most significant, individual p value, one at a time, at each step in the process until no more predictors significant at p≤0.05 remained.

Step 4. From this model we calculated the PP for PD in a tested individual, using the regression coefficient values B obtained from the logistic regression model via the following equation: eN/(1 + eN), wherein N = −0.45+ Σi = 1-n (Bi*ln(Gene_expi)), wherein each i in said formula indicates a different gene i; Bi is the regression coefficient value of said gene i; and Gene_expi is the relative expression level of said gene i in said individual.

Step 5. The PP values were used to construct a ROC curve (and AUC) depicting the relationship between sensitivity and specificity for the early/mild PD group versus healthy controls.

Step 6. A correlation analysis between the individual variables (gene expression levels) was performed to ascertain that these do not highly correlate, as this would add no further resolution to the model. Notably, all the correlations were similar with a maximal R value of 0.592.

### Statistical analysis

To determine if the predicted risk for PD was independent of age, the main risk factor for PD, it was introduced as a possible explanatory variable within the regression model alongside with the gene expression variables. Since the proportion of males was significantly higher in the early PD group (Mann–Whitney non-parametric test, p = 0.012), two-way ANOVA (followed by Tukey post-hoc analysis), with gender (male/female) and group (control/PD) as independent variables was performed.

To assess whether dopamine replacement therapy may influence the prediction for PD risk by the model *t*-test analysis was applied to compare between the PP values of non-medicated, de-novo PD cohort and those of the early medicated population.

Comparison between the experimental groups was carried out using one-way ANOVA followed by Tukey post-hoc analysis. Correlations were evaluated via Pearson Correlation with two tailed test of significance. All statistical analyses were performed using SPSS Statistics 17.0 software (SPSS Inc., Chicago, Illinois, USA).

## Competing interests

MBY received royalties from TEVA pharmaceutical for the development of rasagiline. EG institution received grant funds from Verein Zur Durchfhurung Neurowissensschftlicher Tagungenand and from Hirnliga.

## Authors’ contributions

LM, JMR, MBH and SAM designed the study and prepared the statistical analysis plan. SAM wrote the manuscript with assistance from LM, JMR and MBY. LM, JMR, MBY and SAM contributed to data analyses. YB assisted with technical methods. JMR, ED, UB, RC, DF, EG, PR, CJ and JA identified patients or controls and were responsible for collecting data from them. The authors wish it to be known that, in their opinion, the first 2 authors should be regarded as joint first authors. All authors read and approved the final manuscript.

## Supplementary Material

Additional file 1: Table S1 Hematologic values of PD cases and controls. **Table S2.** Stability ranking of the candidate reference genes, Methods [44,45]. Click here for file

Additional file 2 Run Overview Report, Virtual Gel Report, Egram, Gel Lane And Result Table Report. Click here for file
