# Supplementary material for: A molecular signature in blood identifies early Parkinson’s disease
Source: Mol Neurodegener. 2012 May 31;7:26. doi: 10.1186/1750-1326-7-26 (PMC3424147; doi:10.1186/1750-1326-7-26)
Supplement: Additional file 1: Table S1 — Hematologic values of PD cases and controls. Table S2. Stability ranking of the candidate reference genes, Methods [44,45]. [file 1750-1326-7-26-S1.doc]

**Additional file 1**

**Title: A molecular signature in blood identifies early Parkinson’s disease**

Leonid Molochnikov^1^*****, Jose M Rabey^1,2^*****, Evgenya Dobronevsky^2^, Ubaldo Bonucelli^3,4^, Roberto Ceravolo^4^, Daniela Frosini^4^, Edna Grünblatt^5,6^, Peter Riederer^6^, Christian Jacob^6^, Judith Aharon-Peretz^7^, Yulia Bashenko^8^, Moussa BH Youdim^8,9^ and Silvia A Mandel^8^

^1^Sackler School of Medicine, Tel Aviv University, Tel Aviv, Israel

^2^ Asaf HaRofeh Medical Center, Department of Neurology, Zerifin, Israel

^3^ Neurology Unit, Hospital of Viareggio, Viareggio, Italy

^4^ Department of Neuroscience, University of Pisa, Pisa, Italy

^5^ Neurobiochemistry Laboratory, Department of Child and Adolescent Psychiatry, University Zurich-Irchel, Zurich, Switzerland

^6^Department of Psychiatry, Psychosomatic and Psychotherapy, University Hospital of Würzburg, Würzburg, Germany

^7^ Department of Neurology, Rambam Medical Center, Haifa, Israel

^8^ Technion-Faculty of Medicine, Eve Topf Center for Neurodegenerative Diseases Research, Department of Molecular Pharmacology, Haifa, Israel

^9^Yonsei Central University, Department of Biology, Seoul, Republic of Korea

**Methods**

RNA quality control.

RNA quality was determined spectrophotometrically by NanoDrop 1000 Spectrophotometer (Thermo Fisher Scientific Inc, Wilmington, DE, USA). The OD average ratios derived from RNA spectrometer absorbance for all samples were (average ± SD): 260/280 = 1.95±0.05 and 260/230 = 2.04±0.26. Another quality test included electrophoretic analysis by Experion^TM^ Automated Electrophoresis System (Bio-Rad Laboratories, Hercules, CA, USA). Additional file 2 illustrates the analysis of 12 representative blood RNA samples showing the running pattern and calculated values for RNA area, concentration and ratio of 28S/18S. Rhe RNA quality indicator (RQI) values for all samples ranged from 7.5-9.3 (10=intact RNA; 1=degraded RNA), indicative of high quality.

QRT-PCR

Reactions were primed using QuantiTect Primer Assay, (QIAGEN, Hilden, Germany) and SYBR® Premix Ex Taq™, ROX™ Reference Dye II (Takara, Otsu, Shiga, Japan). The thermal cycler program consisted of an initial denaturation at 95°C for 10 min followed by 40 cycles of denaturation at 95°C for 15 s, and primer annealing at 60°C for 1min. The results were analyzed using 7000 System SDS Software (Applied Biosystems). Baseline values were manually set for each primer to neutralize non-specific background noise and were deduced from the Rn vs. cycle number. Rn is the fluorescence of the reporter dye (sybergreen) divided by the fluorescence of the passive reference dye, ROX. The latter does not participate in the 5' nuclease reaction providing an internal reference for background fluorescence emission. The threshold level of the fluorescence was set to Delta Rn = 0.2 (default threshold level). Raw Ct values (the point at which the fluorescence crosses the threshold) were transformed to quantities automatically using the mentioned software via the equation [Qty=10^((ct-Intercept)/Slope], with Slope and Intercept values taken from the Ct vs. Log Qty standard curve. cDNA dilution series were performed to ascertain that target genes and housekeeping genes have equal amplification efficiencies. Negative control included reaction samples without template or reverse transcriptase. In order to account for inter-assay variations a set of at least 2 reference cDNA samples were run per plate, producing internal positive control (IPC) values. The quantities were then normalized to IPC to control for inter-plate variability.

Reliability of the Housekeeping genes

To ensure correct normalization of the expression levels for the genes of interest, the stability of five housekeeping genes (HKs) in human leukocyte samples, namely ACTB, GAPDH, ALAS1, PPIA and RPL13A was assessed in all PD cohorts (early and advanced) and healthy age matched controls using two specific Visual Basic for Applications (VBA) applets geNorm and NormFinder, to produce the most stable panel of HKs [44,45]. Three most stable internal control genes, GAPDH, ACTB and ALAS1 were selected. GeNorm classified ACTB and ALAS1 as the best two controls of the group, with GAPDH ranking third (Suppl Table 3). The best position in the stability ranking produced by NormFinder was occupied by ACTB, followed by GAPDH and ALAS1. Since the minimal use of three most stable internal control genes for optimal RT-PCR normalization is recommended[45], the three HKs GAPDH, ACTB and ALAS1 were selected. The relative gene expression was calculated by dividing the raw quantities of the gene of interest by the geometric mean of the 3 HKs. The relative gene expression was normalized to the geometric mean of the three most stable internal control HK genes, GAPDH, ACTB and ALAS1.

**Additional Tables**

**Additional Table S1. Hematologic values of PD cases and controls**

| **Diagnostic groups** | **Control** | **PD (total cases)** |
| --- | --- | --- |
| WBC [10^3/µl] (SD) | 7.98 (1.99) | 6.94 (1.74) |
| Neutrophils [10^3/µl] (SD) | 5.10 (2.07) | 4.36 (1.32) |
| Lymphocytes [10^3/µl] (SD) | 2.09 (0.86) | 1.93 (0.76) |
| Monocytes [10^3/µl] (SD) | 0.61 (0.19) | 0.48 (0.15) |
| Eosinophils [10^3/µl] (SD) | 0.16 (0.09) | 0.14 (0.11) |
| Basophils [10^3/µl] (SD) | 0.02 (0.04) | 0.04 (0.04) |
| NRBC [10^3/µl] (SD) | 0.00 (0.00) | 0.00 (0.01) |
| RBC [10^6/µl] (SD) | 4.57 (0.50) | 4.55 (0.48) |
| HGB [g/dl] (SD) | 13.55 (1.32) | 13.84 (1.38) |
| HCT [%] (SD) | 39.86 (3.59) | 41.02 (4.31) |
| MCV [µm^3] (SD) | 87.57 (5.41) | 90.13 (4.58) |
| MCH [pg] (SD) | 29.74 (2.04) | 30.21 (1.75) |
| MCHC [g/dl] (SD) | 33.93 (0.83) | 33.67 (1.16) |
| RDW [%] (SD) | 13.97 (1.34) | 14.36 (1.50) |
| Platelet count [10^3/µl] (SD) | 236.3 (58.5) | 241.2 (54.8) |
| MPV [µm^3] (SD) | 8.52 (1.13) | 8.42 (1.35) |

**Additional Table S2**: Stability ranking of the candidate reference genes

| **Software** | **Ranking** | | | | |
| --- | --- | --- | --- | --- | --- |
|  | 1^st^ | 2^nd^ | 3^rd^ | 4^th^ | 5^th^ |
| geNorm (Average M value) | ACTB & ALAS1* (0.457) | | GAPDH (0.536) | PPIA (0.69) | RPL13A (0.922) |
| NormFinder (Average Stability Value) | ACTB (0.061) | GAPDH (0.133) | ALAS1 (0.229) | RPL13A (0.265) | PPIA (0.275) |
| * means that 1st and 2nd positions cannot be further ranked by geNorm.  Lower values indicate greater stability | | | | | |
