# Supplementary material for: A molecular signature in blood identifies early Parkinson’s disease
Source: Mol Neurodegener. 2012 May 31;7:26. doi: 10.1186/1750-1326-7-26 (PMC3424147; doi:10.1186/1750-1326-7-26)
Supplement: Additional file 2 — Run Overview Report, Virtual Gel Report, Egram, Gel Lane And Result Table Report. [file 1750-1326-7-26-S2.pdf]

Run Overview Report

**Project:**	PGX160511Yulia	**Acq. Analyst:**	DefaultUser
**Assay:**	Eukaryote Total RNA StdSens	**Acq. Time:**	5/16/2011 9:12:23 AM
**Run:**	Run_Eukaryote_TotalRNA_StdSens_001398_5-16-2011_9-12-22 AM	**Signature:**	N/A
**Run Version:**	N/A		

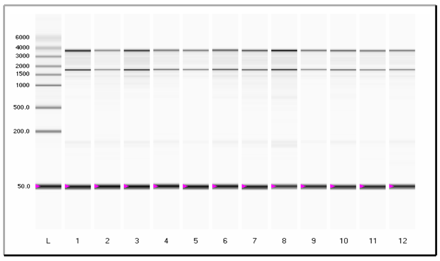

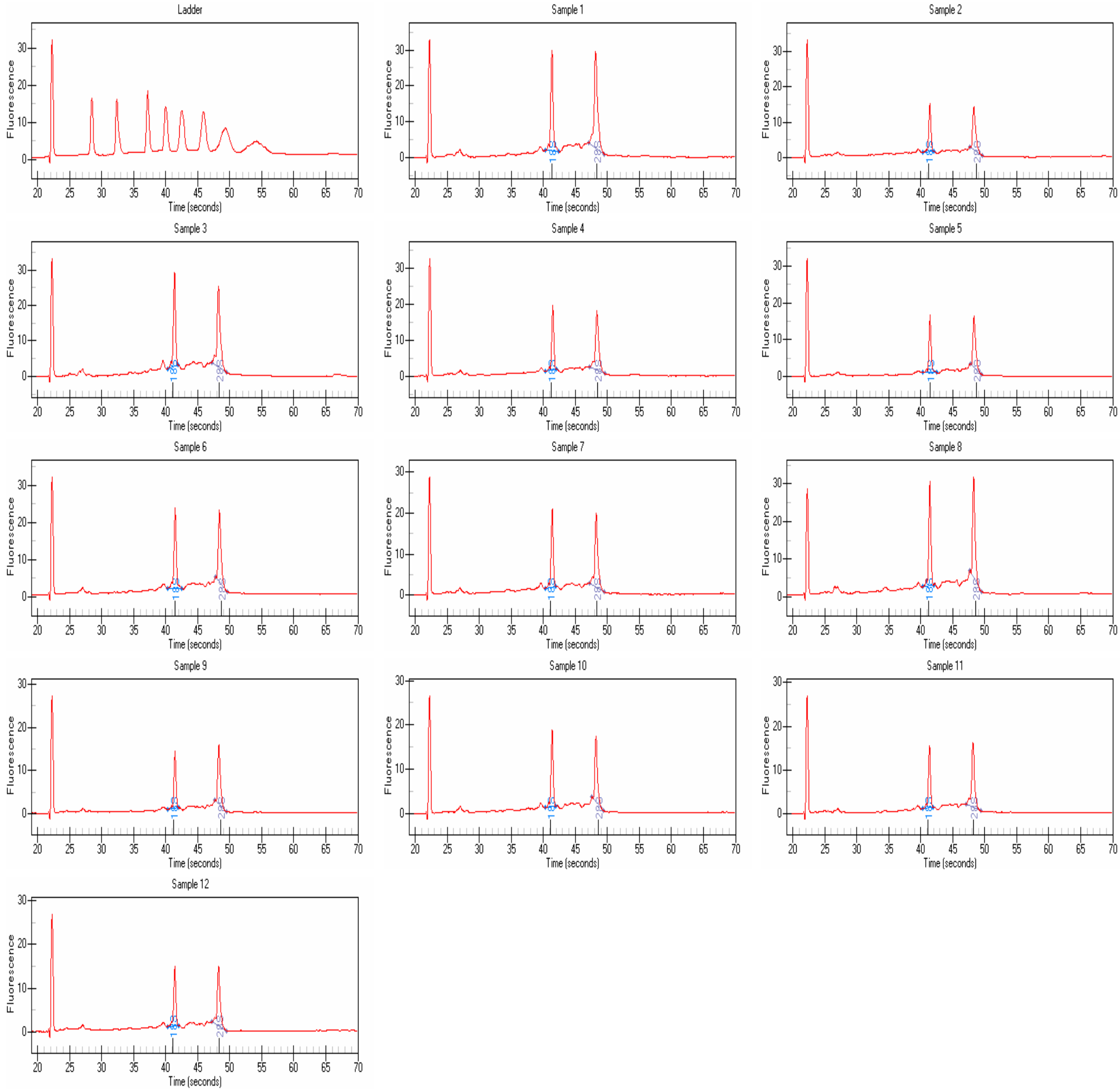

Virtual Gel Report

**Project:**	PGX160511Yulia	**Acq. Analyst:**	DefaultUser
**Assay:**	Eukaryote Total RNA StdSens	**Acq. Time:**	5/16/2011 9:12:23 AM
**Run:**	Run_Eukaryote_TotalRNA_StdSens_001398_5-16-2011_9-12-22 AM	**Signature:**	N/A
**Run Version:**	N/A		

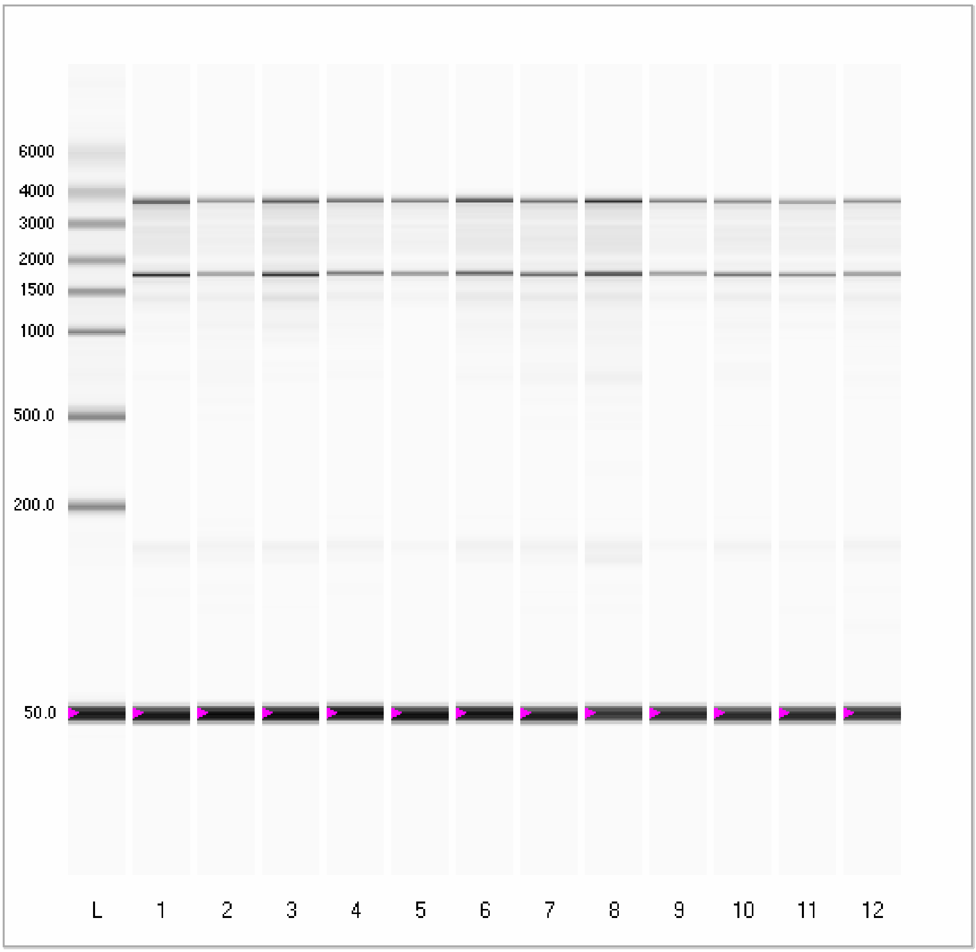

**Project:**	PGX160511Yulia	**Acq. Analyst:**	DefaultUser
**Assay:**	Eukaryote Total RNA StdSens	**Acq. Time:**	5/16/2011 9:12:23 AM
**Run:**	Run_Eukaryote_TotalRNA_StdSens_001398_5-16-2011_9-12-22 AM	**Signature:**	N/A
**Run Version:**	N/A		

Well# Ladder

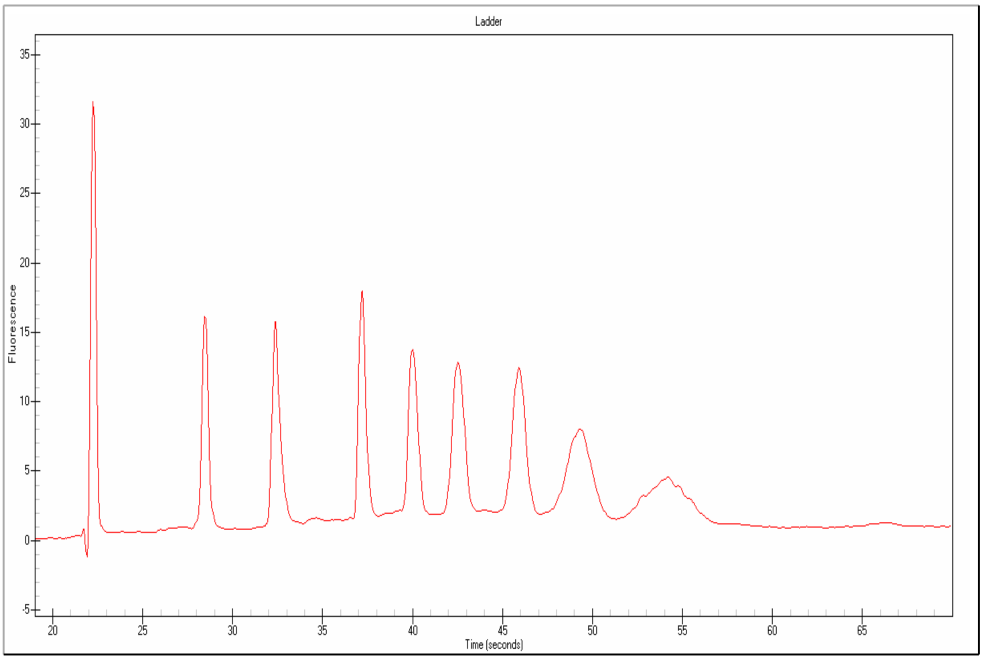

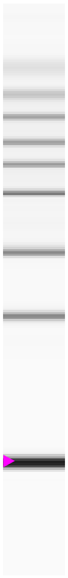

L

Well# Ladder

RNA Area: 242.68

RNA Concentration: 160.00 ng/µl

Well# Ladder

Peak State	Peak Number	Mig. Time (secs)	Corrected Area	Comments
	1	21.75	0.38	
	2	22.25	47.70	
L	3	28.45	22.91	
L	4	32.40	23.15	
L	5	37.20	21.62	
	6	39.30	0.96	
L	7	40.00	19.98	
L	8	42.50	23.19	
	9	43.95	1.59	
L	10	45.90	22.96	

**Project:**	PGX160511Yulia	**Acq. Analyst:**	DefaultUser
**Assay:**	Eukaryote Total RNA StdSens	**Acq. Time:**	5/16/2011 9:12:23 AM
**Run:**	Run_Eukaryote_TotalRNA_StdSens_001398_5-16-2011_9-12-22 AM	**Signature:**	N/A
**Run Version:**	N/A		

Well# Ladder

Peak State	Peak Number	Mig. Time (secs)	Corrected Area	Comments
L	11	49.35	23.96	
	12	52.80	3.04	
	13	54.20	13.80	

Egram, Gel Lane and Result Table Report

**Project:**	PGX160511Yulia	**Acq. Analyst:**	DefaultUser
**Assay:**	Eukaryote Total RNA StdSens	**Acq. Time:**	5/16/2011 9:12:23 AM
**Run:**	Run_Eukaryote_TotalRNA_StdSens_001398_5-16-2011_9-12-22 AM	**Signature:**	N/A
**Run Version:**	N/A		

Well# 1 Sample 1

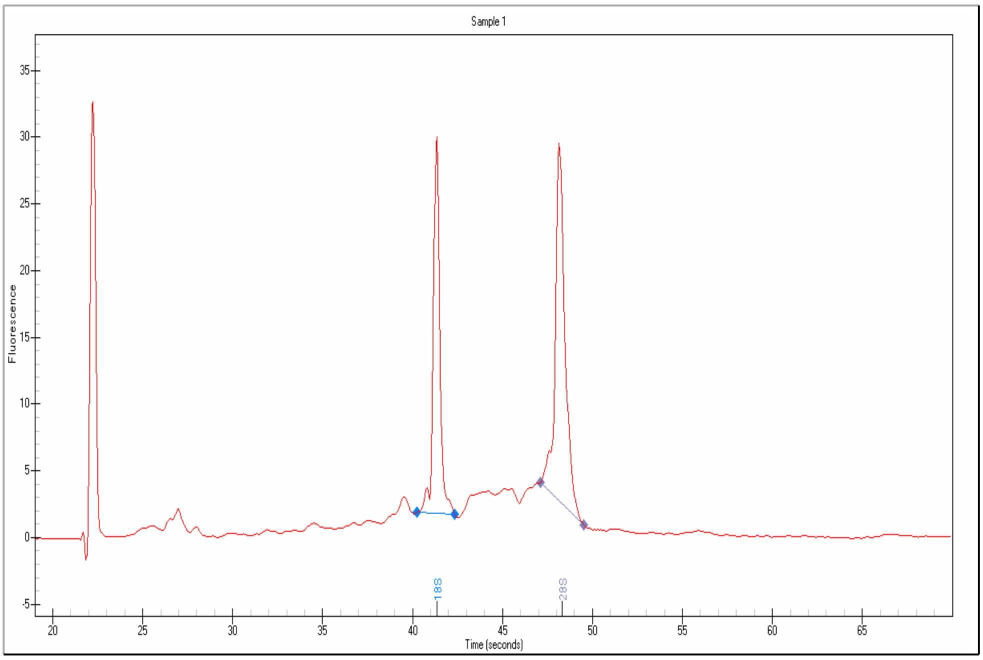

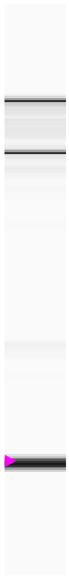

1

Well# 1 Sample 1

Fragment Number	Fragment Name	Start Time	End Time	Area	% of Total Area
1	18S	40.30	42.40	29.20	17.38
2	28S	47.10	49.50	35.09	20.89

RNA Area: 167.97
RNA Concentration: 110.75 ng/µl
Ratio[28S/18S]: 1.20

RQI: 9.3

Well# 1 Sample 1

Peak State	Peak Number	Mig. Time (secs)	Corrected Area	Comments
	1	22.25	49.60	
	2	26.97	2.74	
	3	27.96	0.82	
	4	39.53	2.00	
	5	40.78	0.99	
	6	41.32	25.58	
	7	47.58	4.10	

**Project:**	PGX160511Yulia	**Acq. Analyst:**	DefaultUser
**Assay:**	Eukaryote Total RNA StdSens	**Acq. Time:**	5/16/2011 9:12:23 AM
**Run:**	Run_Eukaryote_TotalRNA_StdSens_001398_5-16-2011_9-12-22 AM	**Signature:**	N/A
**Run Version:**	N/A		

Well# 1 Sample 1

Peak State	Peak Number	Mig. Time (secs)	Corrected Area	Comments
	8	48.18	36.72	

Egram, Gel Lane and Result Table Report

**Project:**	PGX160511Yulia	**Acq. Analyst:**	DefaultUser
**Assay:**	Eukaryote Total RNA StdSens	**Acq. Time:**	5/16/2011 9:12:23 AM
**Run:**	Run_Eukaryote_TotalRNA_StdSens_001398_5-16-2011_9-12-22 AM	**Signature:**	N/A
**Run Version:**	N/A		

Well# 2 Sample 2

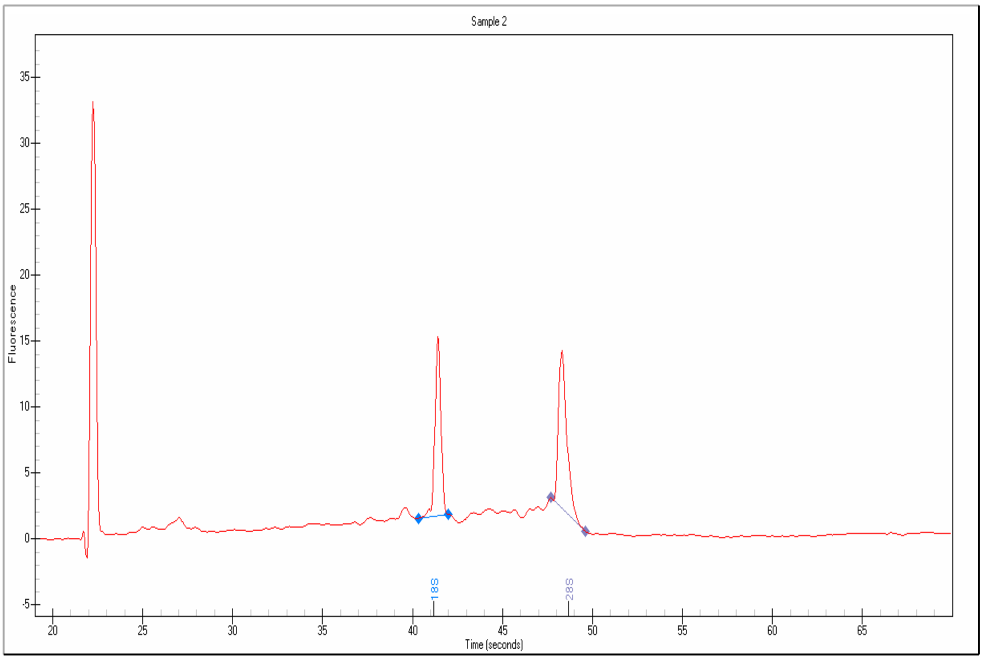

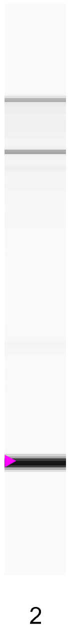

Well# 2 Sample 2

Fragment Number	Fragment Name	Start Time	End Time	Area	% of Total Area
1	18S	40.35	42.00	12.73	11.91
2	28S	47.70	49.60	13.36	12.49

RNA Area:	106.92
RNA Concentration:	70.49 ng/µl
Ratio[28S/18S]:	1.05
RQI:	8.0

Well# 2 Sample 2

Peak State	Peak Number	Mig. Time (secs)	Corrected Area	Comments
	1	22.25	49.14	
	2	27.06	1.11	
	3	39.59	0.90	
	4	40.88	0.36	
	5	41.43	12.37	
	6	46.98	0.82	
	7	47.67	1.61	

**Project:**	PGX160511Yulia	**Acq. Analyst:**	DefaultUser
**Assay:**	Eukaryote Total RNA StdSens	**Acq. Time:**	5/16/2011 9:12:23 AM
**Run:**	Run_Eukaryote_TotalRNA_StdSens_001398_5-16-2011_9-12-22 AM	**Signature:**	N/A
**Run Version:**	N/A		

Well# 2 Sample 2

Peak State	Peak Number	Mig. Time (secs)	Corrected Area	Comments
	8	48.32	16.96	

Egram, Gel Lane and Result Table Report

**Project:**	PGX160511Yulia	**Acq. Analyst:**	DefaultUser
**Assay:**	Eukaryote Total RNA StdSens	**Acq. Time:**	5/16/2011 9:12:23 AM
**Run:**	Run_Eukaryote_TotalRNA_StdSens_001398_5-16-2011_9-12-22 AM	**Signature:**	N/A
**Run Version:**	N/A		

Well# 3 Sample 3

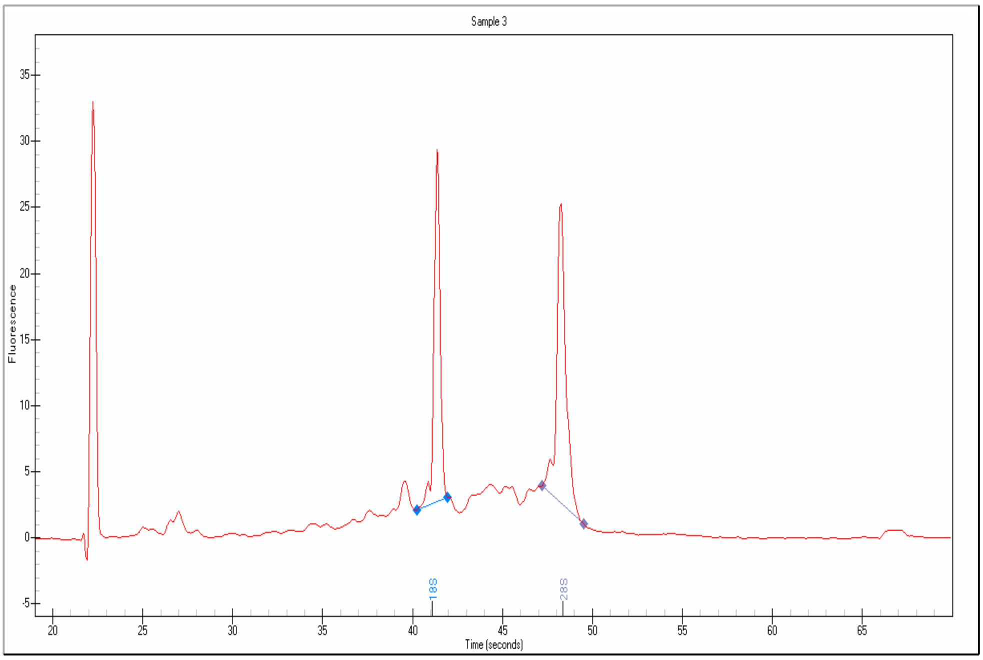

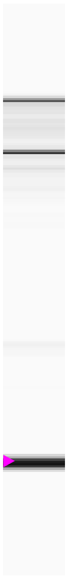

3

**Well# 3 Sample 3**					
Fragment Number	Fragment Name	Start Time	End Time	Area	% of Total Area
1	18S	40.25	41.95	25.46	15.40
2	28S	47.20	49.50	27.63	16.71

RNA Area: 165.38

RNA Concentration: 109.04 ng/µl

Ratio[28S/18S]: 1.09

RQI: 9.1

**Well# 3 Sample 3**				
Peak State	Peak Number	Mig. Time (secs)	Corrected Area	Comments
	1	22.25	49.20	
	2	25.06	0.91	
	3	26.99	2.43	
	4	37.59	0.64	
	5	39.57	4.09	
	6	40.85	2.78	
	7	41.39	29.24	

Project: PGX160511Yulia
Assay: Eukaryote Total RNA StdSens
Run: Run_Eukaryote_TotalRNA_StdSens_001398_5-16-2011_9-12-22 AM
Run Version: N/A

Acq. Analyst: DefaultUser
Acq. Time: 5/16/2011 9:12:23 AM
Signature: N/A

Well# 3 Sample 3

Peak State	Peak Number	Mig. Time (secs)	Corrected Area	Comments
	8	44.35	6.03	
	9	47.66	4.93	
	10	48.25	30.52	

**Project:**	PGX160511Yulia	**Acq. Analyst:**	DefaultUser
**Assay:**	Eukaryote Total RNA StdSens	**Acq. Time:**	5/16/2011 9:12:23 AM
**Run:**	Run_Eukaryote_TotalRNA_StdSens_001398_5-16-2011_9-12-22 AM	**Signature:**	N/A
**Run Version:**	N/A		

Well# 4 Sample 4

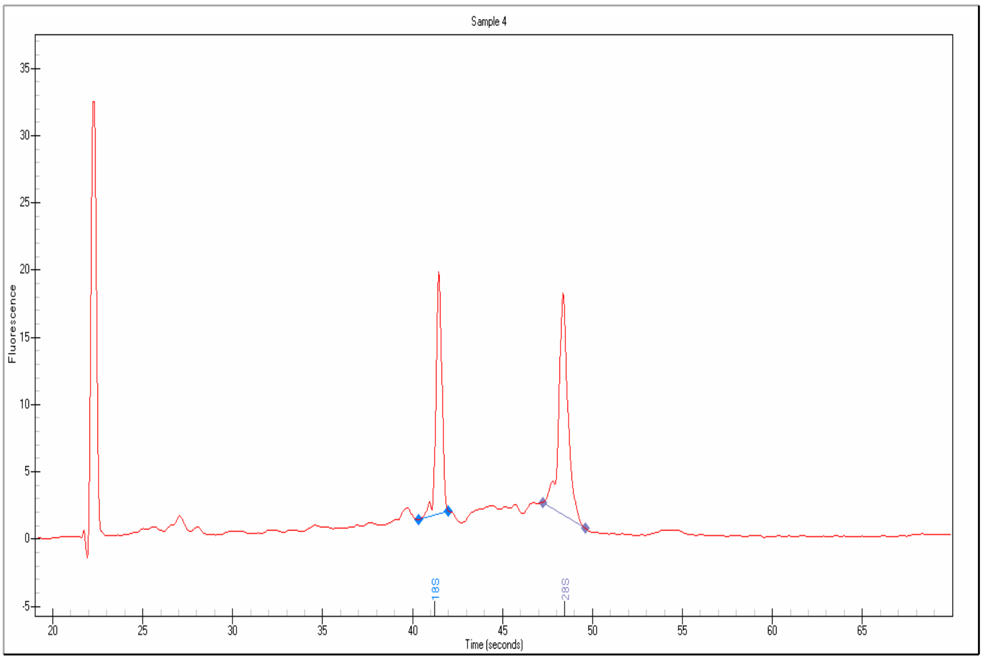

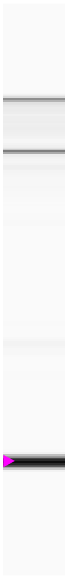

4

Well# 4 Sample 4

Fragment Number	Fragment Name	Start Time	End Time	Area	% of Total Area
1	18S	40.40	42.05	16.96	15.35
2	28S	47.30	49.60	20.16	18.25

RNA Area: 110.45
RNA Concentration: 72.82 ng/µl
Ratio[28S/18S]: 1.19

RQI: 8.7

Well# 4 Sample 4

Peak State	Peak Number	Mig. Time (secs)	Corrected Area	Comments
	1	22.25	48.49	
	2	27.07	1.88	
	3	39.68	1.47	
	4	40.96	0.84	
	5	41.45	16.42	
	6	47.80	1.48	
	7	48.34	19.13	

Egram, Gel Lane and Result Table Report

**Project:**	PGX160511Yulia	**Acq. Analyst:**	DefaultUser
**Assay:**	Eukaryote Total RNA StdSens	**Acq. Time:**	5/16/2011 9:12:23 AM
**Run:**	Run_Eukaryote_TotalRNA_StdSens_001398_5-16-2011_9-12-22 AM	**Signature:**	N/A
**Run Version:**	N/A		

Well# 5 Sample 5

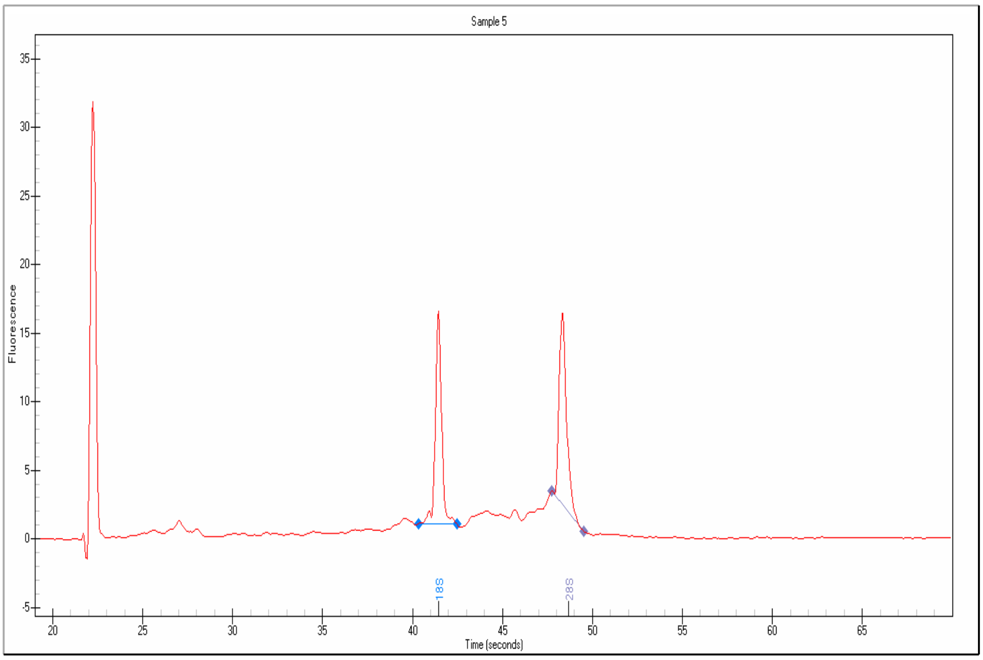

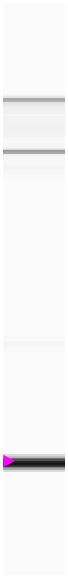

5

**Well# 5 Sample 5**					
Fragment Number	Fragment Name	Start Time	End Time	Area	% of Total Area
1	18S	40.40	42.45	15.01	16.54
2	28S	47.75	49.55	13.75	15.16

RNA Area: 90.74
RNA Concentration: 59.83 ng/µl
Ratio[28S/18S]: 0.92

RQI: 8.4

**Well# 5 Sample 5**				
**Peak State**	**Peak Number**	**Mig. Time (secs)**	**Corrected Area**	**Comments**
	1	22.25	47.37	
	2	27.05	1.72	
	3	40.92	0.54	
	4	41.46	13.64	
	5	45.68	0.35	
	6	47.78	2.45	
	7	48.32	18.04	

Egram, Gel Lane and Result Table Report

**Project:**	PGX160511Yulia	**Acq. Analyst:**	DefaultUser
**Assay:**	Eukaryote Total RNA StdSens	**Acq. Time:**	5/16/2011 9:12:23 AM
**Run:**	Run_Eukaryote_TotalRNA_StdSens_001398_5-16-2011_9-12-22 AM	**Signature:**	N/A
**Run Version:**	N/A		

Well# 6 Sample 6

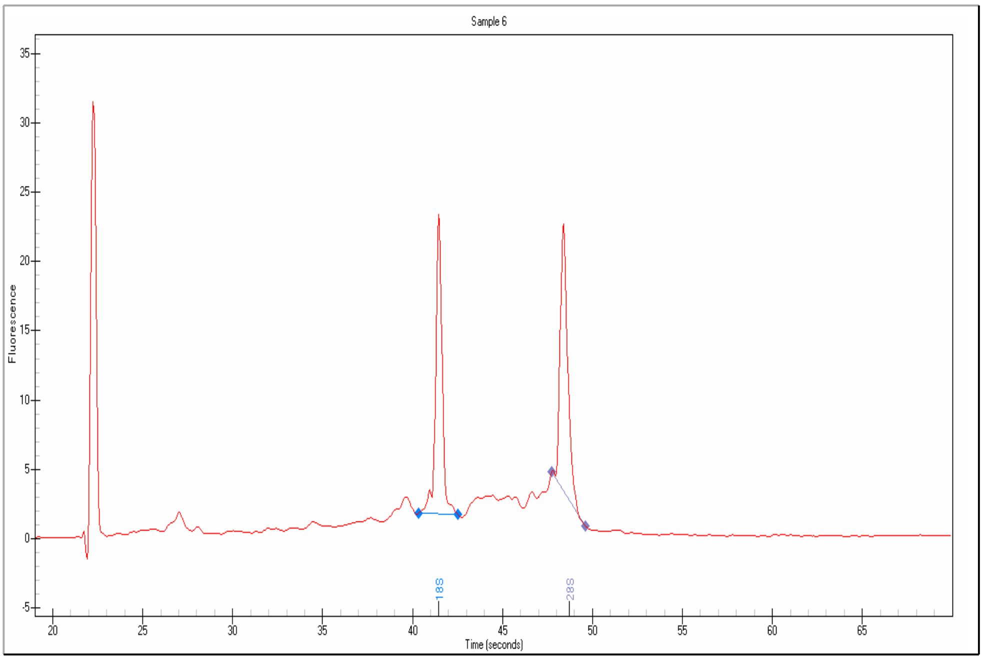

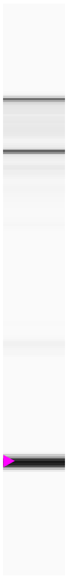

6

Well# 6 Sample 6

Fragment Number	Fragment Name	Start Time	End Time	Area	% of Total Area
1	18S	40.40	42.50	21.78	16.05
2	28S	47.80	49.60	18.82	13.87

RNA Area: 135.67
RNA Concentration: 89.45 ng/µl
Ratio[28S/18S]: 0.86

RQI: 7.8

Well# 6 Sample 6

Peak State	Peak Number	Mig. Time (secs)	Corrected Area	Comments
	1	22.25	46.14	
	2	27.05	2.44	
	3	39.65	1.89	
	4	40.92	0.84	
	5	41.46	19.12	
	6	46.66	0.71	
	7	47.78	2.52	

Egram, Gel Lane and Result Table Report

**Project:**	PGX160511Yulia	**Acq. Analyst:**	DefaultUser
**Assay:**	Eukaryote Total RNA StdSens	**Acq. Time:**	5/16/2011 9:12:23 AM
**Run:**	Run_Eukaryote_TotalRNA_StdSens_001398_5-16-2011_9-12-22 AM	**Signature:**	N/A
**Run Version:**	N/A		

Well# 6 Sample 6

Peak State	Peak Number	Mig. Time (secs)	Corrected Area	Comments
	8	48.37	24.17	

Project: PGX160511Yulia
Assay: Eukaryote Total RNA StdSens
Run: Run_Eukaryote_TotalRNA_StdSens_001398_5-16-2011_9-12-22 AM
Run Version: N/A
Acq. Analyst: DefaultUser
Acq. Time: 5/16/2011 9:12:23 AM
Signature: N/A

Well# 7 Sample 7

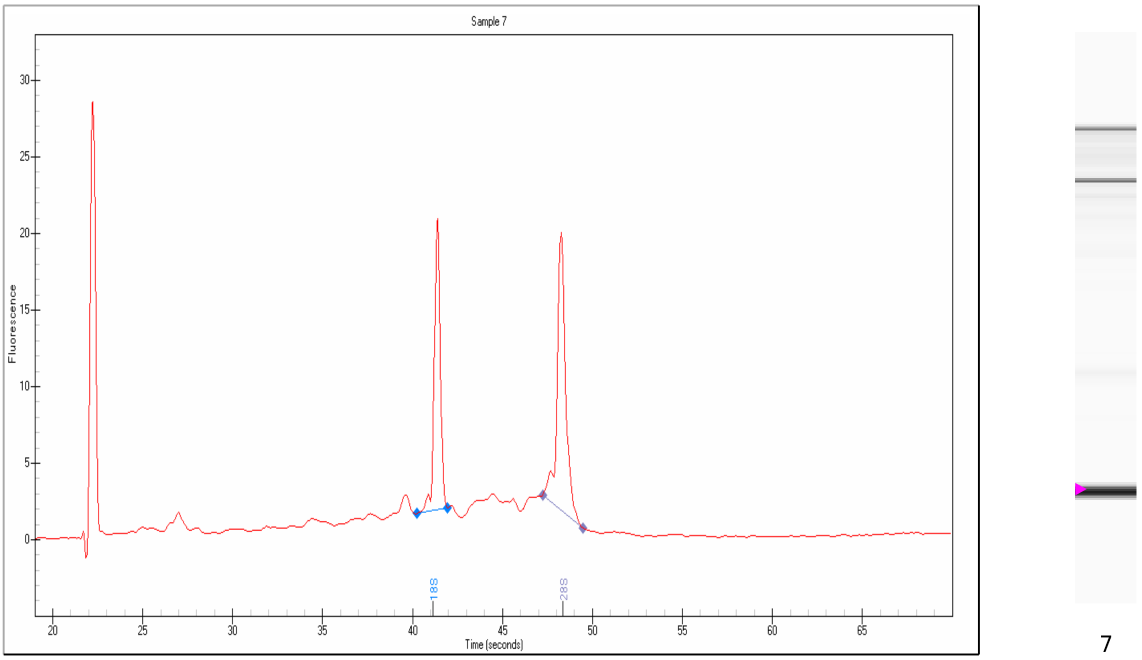

Well# 7 Sample 7					
Fragment Number	Fragment Name	Start Time	End Time	Area	% of Total Area
1	18S	40.30	41.95	17.86	14.67
2	28S	47.25	49.45	20.48	16.82

RNA Area: 121.75
RNA Concentration: 80.27 ng/µl
Ratio[28S/18S]: 1.15

RQI: 8.4

Well# 7 Sample 7				
Peak State	Peak Number	Mig. Time (secs)	Corrected Area	Comments
	1	22.25	41.77	
	2	27.00	1.83	
	3	39.60	1.65	
	4	40.87	0.71	
	5	41.36	16.96	
	6	47.69	1.70	
	7	48.27	19.42	

Egram, Gel Lane and Result Table Report

**Project:**	PGX160511Yulia	**Acq. Analyst:**	DefaultUser
**Assay:**	Eukaryote Total RNA StdSens	**Acq. Time:**	5/16/2011 9:12:23 AM
**Run:**	Run_Eukaryote_TotalRNA_StdSens_001398_5-16-2011_9-12-22 AM	**Signature:**	N/A
**Run Version:**	N/A		

Well# 8 Sample 8

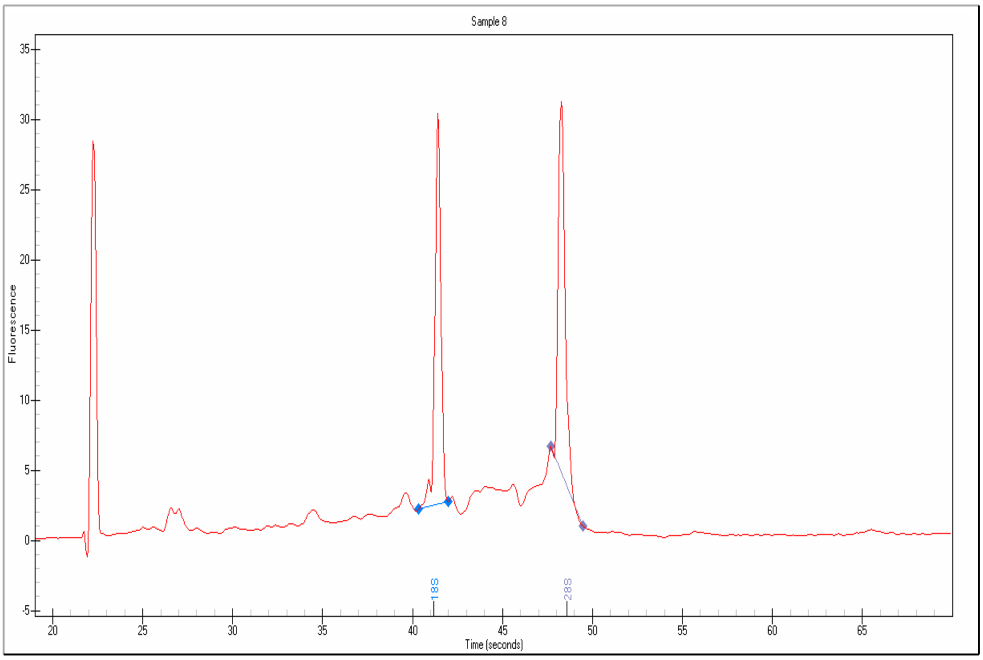

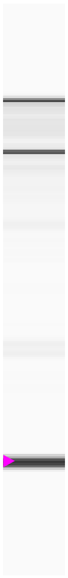

8

**Well# 8 Sample 8**					
Fragment Number	Fragment Name	Start Time	End Time	Area	% of Total Area
1	18S	40.35	42.00	26.13	15.76
2	28S	47.70	49.45	23.94	14.43

RNA Area: 165.83
RNA Concentration: 109.34 ng/µl
Ratio[28S/18S]: 0.92

RQI: 7.7

**Well# 8 Sample 8**				
**Peak State**	**Peak Number**	**Mig. Time (secs)**	**Corrected Area**	**Comments**
	1	22.25	40.88	
	2	26.58	2.06	
	3	27.02	1.98	
	4	34.46	1.82	
	5	39.63	1.71	
	6	40.91	1.04	
	7	41.40	24.32	

**Project:**	PGX160511Yulia	**Acq. Analyst:**	DefaultUser
**Assay:**	Eukaryote Total RNA StdSens	**Acq. Time:**	5/16/2011 9:12:23 AM
**Run:**	Run_Eukaryote_TotalRNA_StdSens_001398_5-16-2011_9-12-22 AM	**Signature:**	N/A
**Run Version:**	N/A		

Well# 8 Sample 8

Peak State	Peak Number	Mig. Time (secs)	Corrected Area	Comments
	8	47.70	2.64	
	9	48.24	29.31	

**Project:**	PGX160511Yulia	**Acq. Analyst:**	DefaultUser
**Assay:**	Eukaryote Total RNA StdSens	**Acq. Time:**	5/16/2011 9:12:23 AM
**Run:**	Run_Eukaryote_TotalRNA_StdSens_001398_5-16-2011_9-12-22 AM	**Signature:**	N/A
**Run Version:**	N/A		

Well# 9 Sample 9

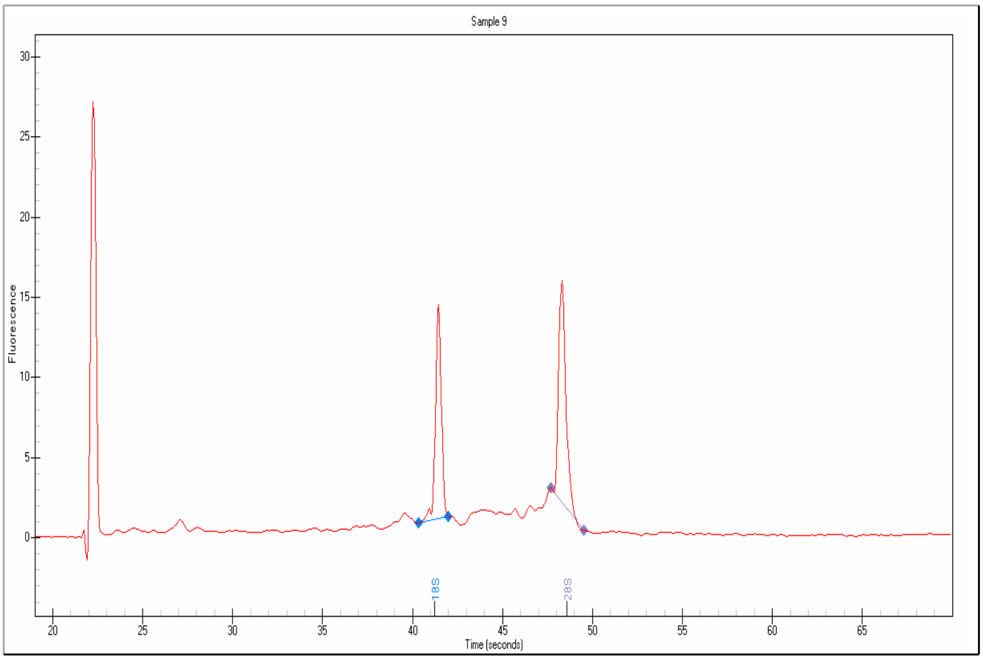

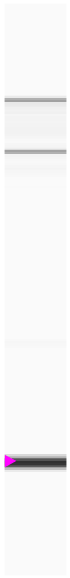

9

Well# 9 Sample 9

Fragment Number	Fragment Name	Start Time	End Time	Area	% of Total Area
1	18S	40.40	42.05	12.50	15.73
2	28S	47.70	49.50	13.31	16.74

RNA Area: 79.50
RNA Concentration: 52.42 ng/µl
Ratio[28S/18S]: 1.06

RQI: 8.8

Well# 9 Sample 9

Peak State	Peak Number	Mig. Time (secs)	Corrected Area	Comments
	1	22.25	38.96	
	2	27.05	1.02	
	3	39.61	1.06	
	4	40.89	0.44	
	5	41.43	11.85	
	6	46.53	1.29	
	7	47.71	2.29	

Egram, Gel Lane and Result Table Report

Project: PGX160511Yulia
Assay: Eukaryote Total RNA StdSens
Run: Run_Eukaryote_TotalRNA_StdSens_001398_5-16-2011_9-12-22 AM
Run Version: N/A
Acq. Analyst: DefaultUser
Acq. Time: 5/16/2011 9:12:23 AM
Signature: N/A

Well# 9 Sample 9

Peak State	Peak Number	Mig. Time (secs)	Corrected Area	Comments
	8	48.31	16.80	

Project: PGX160511Yulia
Assay: Eukaryote Total RNA StdSens
Run: Run_Eukaryote_TotalRNA_StdSens_001398_5-16-2011_9-12-22 AM
Run Version: N/A
Acq. Analyst: DefaultUser
Acq. Time: 5/16/2011 9:12:23 AM
Signature: N/A

Well# 10 Sample 10

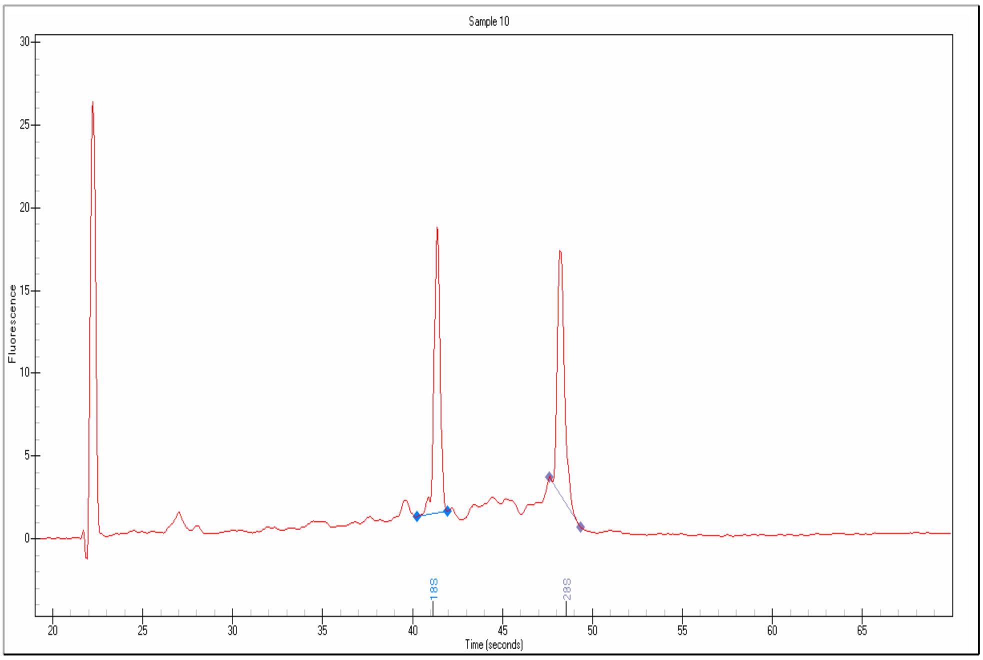

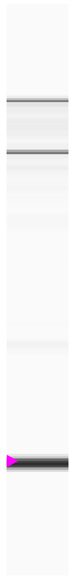

10

Well# 10 Sample 10

Fragment Number	Fragment Name	Start Time	End Time	Area	% of Total Area
1	18S	40.30	41.95	16.40	16.13
2	28S	47.65	49.40	13.81	13.58

RNA Area: 101.67
RNA Concentration: 67.03 ng/µl
Ratio[28S/18S]: 0.84

RQI: 7.5

Well# 10 Sample 10

Peak State	Peak Number	Mig. Time (secs)	Corrected Area	Comments
	1	22.25	38.36	
	2	27.00	3.03	
	3	39.61	1.40	
	4	40.84	0.63	
	5	41.39	15.63	
	6	47.66	1.51	
	7	48.21	16.88	

Egram, Gel Lane and Result Table Report

**Project:**	PGX160511Yulia	**Acq. Analyst:**	DefaultUser
**Assay:**	Eukaryote Total RNA StdSens	**Acq. Time:**	5/16/2011 9:12:23 AM
**Run:**	Run_Eukaryote_TotalRNA_StdSens_001398_5-16-2011_9-12-22 AM	**Signature:**	N/A
**Run Version:**	N/A		

Well# 11 Sample 11

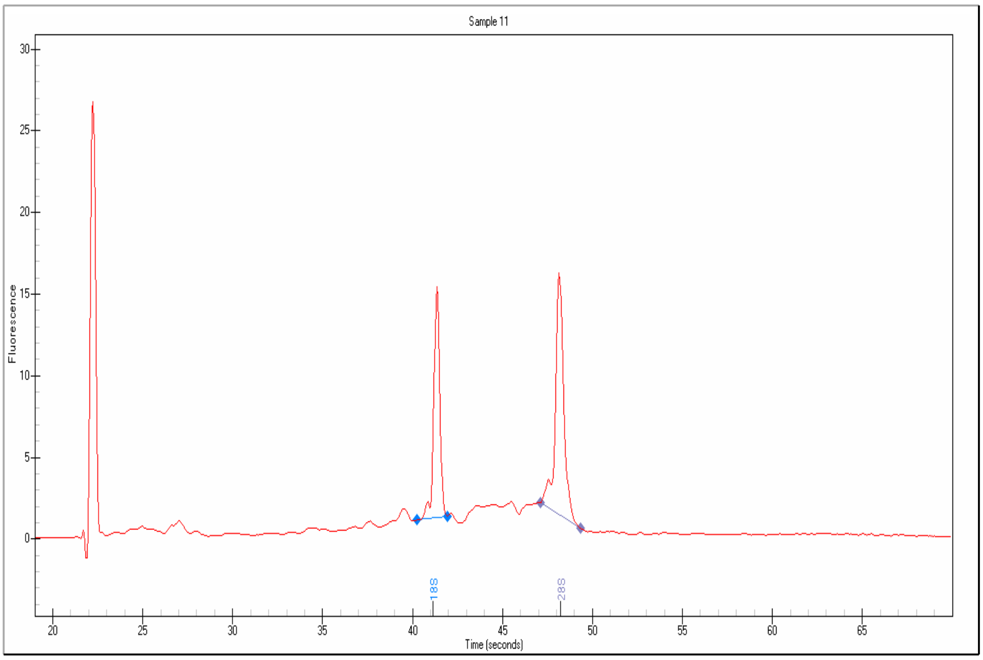

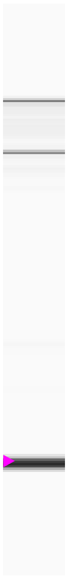

11

**Well# 11 Sample 11**					
Fragment Number	Fragment Name	Start Time	End Time	Area	% of Total Area
1	18S	40.30	41.95	13.30	14.69
2	28S	47.10	49.35	16.79	18.55

RNA Area:	90.52
RNA Concentration:	59.68 ng/µl
Ratio[28S/18S]:	1.26
RQI:	9.0

**Well# 11 Sample 11**				
**Peak State**	**Peak Number**	**Mig. Time (secs)**	**Corrected Area**	**Comments**
	1	22.25	38.76	
	2	27.02	1.12	
	3	39.53	1.06	
	4	40.82	0.66	
	5	41.37	12.49	
	6	47.58	1.40	
	7	48.18	15.76	

Project: PGX160511Yulia
Assay: Eukaryote Total RNA StdSens
Run: Run_Eukaryote_TotalRNA_StdSens_001398_5-16-2011_9-12-22 AM
Run Version: N/A
Acq. Analyst: DefaultUser
Acq. Time: 5/16/2011 9:12:23 AM
Signature: N/A

Well# 12 Sample 12

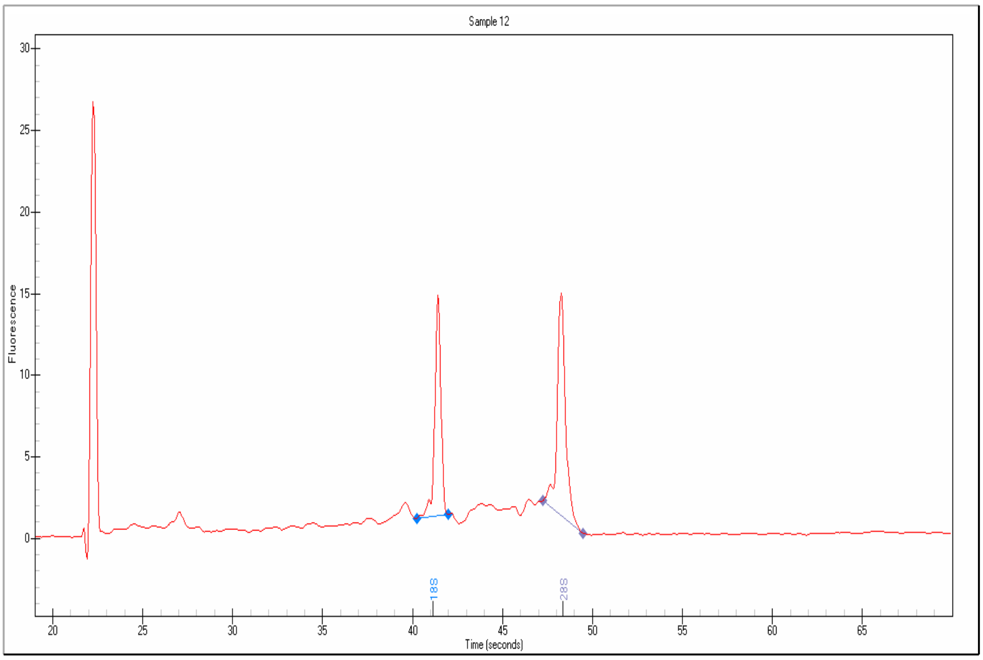

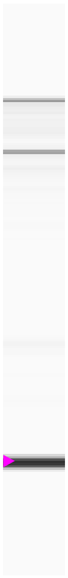

12

Well# 12 Sample 12					
Fragment Number	Fragment Name	Start Time	End Time	Area	% of Total Area
1	18S	40.30	42.00	12.98	14.35
2	28S	47.25	49.45	15.28	16.89

RNA Area: 90.44
RNA Concentration: 59.63 ng/µl
Ratio[28S/18S]: 1.18

RQI: 8.4

Well# 12 Sample 12				
Peak State	Peak Number	Mig. Time (secs)	Corrected Area	Comments
	1	22.25	38.22	
	2	27.08	1.58	
	3	39.62	1.34	
	4	40.92	0.51	
	5	41.41	11.91	
	6	46.49	1.05	
	7	47.69	2.22	

Egram, Gel Lane and Result Table Report

Project: PGX160511Yulia
Assay: Eukaryote Total RNA StdSens
Run: Run_Eukaryote_TotalRNA_StdSens_001398_5-16-2011_9-12-22 AM
Run Version: N/A

Acq. Analyst: DefaultUser
Acq. Time: 5/16/2011 9:12:23 AM
Signature: N/A

Well# 12 Sample 12

Peak State	Peak Number	Mig. Time (secs)	Corrected Area	Comments
	8	48.23	15.88	
